# Insomnia as a Partial Mediator of the Relationship Between Personality and Future Symptoms of Anxiety and Depression Among Nurses

**DOI:** 10.3389/fpsyg.2019.00901

**Published:** 2019-04-26

**Authors:** Torhild Anita Sørengaard, Håvard Rudi Karlsen, Eva Langvik, Ståle Pallesen, Bjørn Bjorvatn, Siri Waage, Bente Elisabeth Moen, Ingvild Saksvik-Lehouillier

**Affiliations:** ^1^Department of Psychology, Norwegian University of Science and Technology, Trondheim, Norway; ^2^Department of Psychosocial Science, Faculty of Psychology, University of Bergen, Bergen, Norway; ^3^Department of Global Public Health and Primary Care, University of Bergen, Bergen, Norway

**Keywords:** personality, neuroticism, insomnia, anxiety, depression, mental health, partial mediation

## Abstract

**Background:** This study investigates insomnia as a partial mediator in the relationship between personality and symptoms of anxiety and depression.

**Methods:** The study is based on partly longitudinal data from the ongoing cohort study “Survey of Shift work, Sleep, and Health” (SUSSH) among Norwegian nurses, a survey examining the work situation and health status of Norwegian nurses measured with annual questionnaires. The present study uses data collected in 2012 (Wave 4), 2013 (Wave 5), and 2014 (Wave 6). The final sample at Wave 6 consisted of 2002 participants, of which 91% were females. The questionnaires included items measuring, among others, demographic variables, work time schedule, insomnia (Bergen Insomnia Scale), personality (Mini-IPIP) and anxiety and depression (Hospital Anxiety and Depression Scale).

**Results:** Extraversion and conscientiousness had no significant direct or indirect association with insomnia, anxiety or depression. Neuroticism and insomnia had direct associations to future symptoms of anxiety and depression. Insomnia was also a significant partial mediator of the relationship between both neuroticism and anxiety, and neuroticism and depression, meaning that neuroticism had an indirect relation to symptoms of anxiety and depression through insomnia. When adjusting for previous symptoms of anxiety and depression at Wave 5, insomnia was no longer a significant mediator between neuroticism and anxiety, and only marginally mediated the relationship between neuroticism and depression.

**Conclusion:** The results showed that insomnia may act as a mediator between neuroticism and symptoms of anxiety and depression, but the indirect relationship between neuroticism and anxiety and depression through insomnia is considerably weaker than the direct association. Hence, the mediating effect of insomnia should be interpreted with caution. The sample mainly consisted of female nurses, and the generalizability of the findings to male dominated occupations is limited. Findings from the present study highlight the importance of an integrated approach and strengthen the understanding of how personality and psychopathology are connected.

## Introduction

Anxiety and depression are two of the most common health problems in today’s society (Knudsen et al., 2013). The role of individual dispositional factors (e.g., neuroticism) in the development of these mental disorders is well established (Bienvenu and Stein, 2003; Bienvenu et al., 2004; Klein et al., 2011). Although a high score on certain personality traits may increase the vulnerability of developing anxiety and depression, there is no clear understanding of which pathways or complex mechanisms that can explain the association between personality and mood disorders (Klein et al., 2011). Several variables may influence, and partly or fully mediate this relationship. One prominent variable in this regard is sleep (Huang et al., 2016). Sleep problems represent key symptoms of anxiety and depression (5th ed.; DSM-5, American Psychiatric Association, 2013), and are significant predictors of future symptoms of anxiety (Neckelmann et al., 2007; Vedaa et al., 2016a) and depression (Riemann and Voderholzer, 2003; Baglioni et al., 2011). There is limited knowledge about the relations between personality and healthy sleep patterns (van de Laar et al., 2010; Duggan et al., 2014). However, as sleep and personality independently predict adverse health outcomes (Norlander et al., 2005; Czeisler, 2015; Strickhouser et al., 2017), both may be particularly important in the development of mental disorders and should thus be investigated applying an integrated approach. There is a strong need for identifying both protective and risk factors of development of anxiety and depression at the individual level. This includes investigation of direct and indirect associations between individual differences, e.g., personality, and future symptoms of anxiety and depression.

The relationship between personality and psychopathology is complex. Several models trying to elucidate this relationship include the pathoplastic model (i.e., a bidirectional relationship between personality and psychopathology), the spectrum model (i.e., personality and psychopathology exist on a common spectrum of functioning), and the more common etiological model where personality is assumed to contribute to the onset of mental disorders (Widiger, 2011). In agreement with the latter model certain personality traits, e.g., extraversion and neuroticism, have been shown to play an important role in the development of anxiety and depression (Clark et al., 1994; Bienvenu and Stein, 2003; Muris et al., 2004). While neuroticism is a general distress factor related to a wide range of symptoms and negative affect, extroversion is specifically related to positive affect (Langvik et al., 2016) and inversely to certain emotional disorders like major depression (Watson and Naragon-Gainey, 2014). Conscientiousness is associated with significantly reduced likelihood of a wide range of mental disorders (Goodwin and Friedman, 2006; Hampson et al., 2015), and has shown consistent positive relations to health promoting behavior and longevity (Bogg and Roberts, 2012).

Personality seems to influence subjective quality and timing of sleep, but not necessarily sleep duration (Soehner et al., 2007). Individuals with high scores on neuroticism report more sleep problems and reduced sleep quality compared to those with low scores, whereas high scores on conscientiousness and extraversion are associated with good sleep (Hintsanen et al., 2014). Higher extraversion is associated with less sleep problems (Hintsanen et al., 2014), better sleep quality (Gray and Watson, 2002), and shorter sleep latency (Williams and Moroz, 2009) compared to low extraversion. Further, high scores on neuroticism and low scores on conscientiousness are related to poor sleep quality and increased sleepiness (Duggan et al., 2014). Neuroticism might lead to disrupted sleep through an increase in stress-reactivity (Harvey et al., 2014) and poor sleep hygiene (Mastin et al., 2005). Further, Gurtman et al. (2014) suggest that neuroticism may influence sleep-related cognitive distortions and pre-sleep arousal levels. In studies using polysomnography, individuals high in neuroticism, especially on the facet anxiety, take longer time to fall asleep and spend less time in deep sleep stages (Fuller et al., 1997).

Personality traits may as such act as protective or predisposing, as well as perpetuating factors for insomnia (van de Laar et al., 2010), which in turn may affect the future development and onset of anxiety and depression. While previous research has identified personality and sleep as risk factors for depression, these associations have mostly been examined separately and without taking plausible mediating effects into account (Huang et al., 2016). Huang et al. (2016) found that sleep quality partially mediated the relationship between neuroticism and conscientiousness and depressive symptoms. Insomnia, which is closely related to poor sleep quality, can potentially act as a mediator between personality and future symptoms of anxiety and depression, but this possible link has received little attention in the literature so far.

In addition to direct associations, certain personality traits may influence anxiety and depression through their impact on insomnia. Individuals with high scores on neuroticism have an increased risk for experiencing symptoms of insomnia compared to low-scorers (Gurtman et al., 2014), thereby they may also be more vulnerable to subsequent sleep-related effects on positive and negative affect. Insomnia is suggested to disrupt the reward-related brain function, and hence, contribute to the development of depression (Casement et al., 2016). As personality traits are differentially associated with reinforcement sensitivity (Fuentes et al., 2012), insomnia might be a possible link in understanding the relationship between personality and mood disorders. High scores on conscientiousness can, on the other hand, contribute to healthy sleep patterns, earlier bed- and rise times, and good sleep hygiene (Gray and Watson, 2002), perhaps due to the tendency of high-scorers to be self-disciplined and orderly. In turn, these stable behavioral patterns can contribute to obtainment of sufficient sleep and reduced likelihood of insomnia, and as a positive consequence also reduced prevalence of symptoms of anxiety and depression due to chronic sleep deprivation and poor sleep quality.

We argue that these relationships are especially important to investigate among nurses. Nurses are at particular risk of developing anxiety, depression and sleep problems, as they face several physical and emotional challenges in terms of high work load (Lim et al., 2010), shift work (Lin et al., 2014), long working hours (Stimpfel et al., 2012), and high work demands (Jourdain and Chênevert, 2010). Some personality traits, especially hardiness, has been shown to predict certain aspects of shift work tolerance among nurses (Saksvik-Lehouillier et al., 2012). However, constant exposure to work conditions that negatively influence sleep, can make nurses especially vulnerable to develop mental health problems despite a resilient personality.

Conclusions about the complex relationships between sleep disturbances, anxiety and depression has previously been somewhat difficult to draw due to small and heterogenic samples and an overreliance on cross-sectional studies. Tracing paths between personality and anxiety and depression through potential mediators can help explain the processes involved in the onset and course of mood disorders. This can have implications for treatment and identifying individuals at risk for psychopathology (Klein et al., 2011) and for society at large, for example in terms of economics costs of sick leave.

The present study investigates insomnia as a mediating factor between personality and anxiety and depression using a partly longitudinal design. The use of longitudinal data answers the call for more comprehensive research on the relationship between personality traits, anxiety and depression (Muris et al., 2005), including the linkage between personality and insomnia (van de Laar et al., 2010), and between personality and anxiety and depression through sleep.

**FIGURE 1:**
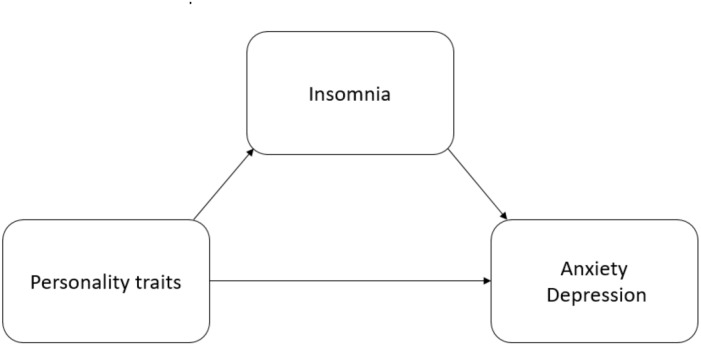
Hypothesized model of direct and indirect relationships between the latent variables.

Figure 1 shows the hypothesized model of direct and indirect relationships between the latent variables. Based on the literature presented in the introduction, we propose the following hypotheses:

1. Neuroticism will be positively related to future symptoms of insomnia, whereas extraversion and conscientiousness will be negatively related to future symptoms of insomnia.
2. Neuroticism and insomnia will be positively related to future symptoms of anxiety and depression, whereas extraversion and conscientiousness will be negatively related to future symptoms of anxiety and depression.
3. Insomnia will partially mediate the relationship between personality and future symptoms of anxiety and depression, also when controlling for previous symptoms of anxiety and depression.

## Materials and Methods

### Design

The present study is based on longitudinal data from the ongoing cohort study “Survey of Shift work, Sleep and Health” (SUSSH) among Norwegian nurses. This survey examines the work situation and health status of Norwegian nurses measured annually. The first wave of data collection was conducted in 2008–2009 (Wave 1), with subsequent data collection taking place annually starting from 2010 (Wave 2). The present study uses data collected in 2012 (Wave 4; personality traits of the Five Factor Model), 2013 (Wave 5; symptoms of insomnia, anxiety and depression), and 2014 (Wave 6; symptoms of anxiety and depression), respectively.

### Ethics Statement

The project was approved by the Regional Committee for Medical Research Ethics (REK) of Western Norway (REK-West, No. 088.08). Informed written consent was obtained from all who participated in the study.

### Procedure

Nurses were enrolled from the member registry of the Norwegian Nurses Organization (NNO). To be eligible for SUSSH, nurses had to be registered in NNO and work at least half-time. A survey sample (*N* = 6000) comprised of five strata, each containing 1200 nurses maintaining at least a 50% work position, was randomly selected from the member register of the NNO. The criteria for the different strata were time elapsed since graduation, in this case 0–11 months (stratum 1), 1–3 years (stratum 2), 3.1–6 years (stratum 3), 6.1–9 years (stratum 4), and 9.1–12 years (stratum 5). The nurses were invited to participate in the SUSSH survey and received the baseline SUSSH questionnaire in the period November 2008 to March 2009. The nurses received paper formatted questionnaires by postal mail and sent them back by prepaid return envelopes. Up to two reminders were sent to nurses who did not respond. A total of 600 invitations were returned due to unknown addresses, which resulted in 5,400 invited nurses of whom 2,059 nurses (response rate: 38%) returned the baseline SUSSH questionnaire. The instruments included in each questionnaire varied. However, many were repeated in each wave.

### Sample

The sample at Wave 6 consisted of 1815 (91%) females and 174 (9%) men. The mean age was 38 years (*SD* = 8.3). Of the 1668 participants who answered the specific question at Wave 6, 331 (20%) worked daytime only, 534 (32%) worked both evening and daytime, 108 (7%) night time only, 620 (37%) worked rotating shift (day, evening, and night time), and 68 (4%) had other work time schedules with both day and night time. For each wave, the same nurses who replied at the first wave were invited to participate again. For Wave 4 the response rate was 76% (*N* = 2172), in Wave 5 the response rate was 69% (*N* = 1923), and lastly 71% in Wave 6 (*N* = 2002).

### Measures

The questionnaires included items measuring (among others) demographic variables, work time schedule, insomnia, personality, anxiety, and depression.

#### Personality

Personality was assessed at Wave 4 using a Norwegian version of the Mini-IPIP, a 20-item short form of the 50-item International Personality Item Pool-Five-Factor Model measure (Goldberg, 1999). The scale is designed to provide a brief assessment of the Five Factor Model (FFM) of personality. It has proven to be a useful, easy to administer and effective tool to measure personality according to the FFM (Donnellan et al., 2006). The MINI-IPIP measures each of the Five Factor Model-traits; neuroticism, extraversion, agreeableness, conscientiousness, and openness to experience, with four items. The respondents are asked to indicate to which degree they identify with a statement like “Talk to a lot of different people at parties.” The responses are provided on a 5-point Likert-type scale ranging from 1 (strongly agree) to 5 (strongly disagree) with a neutral midpoint at 3 (neither agree nor disagree). The Raykov’s reliability coefficient (RRC) was acceptable for neuroticism (0.73) and extraversion (0.78), but the internal consistency was questionable for conscientiousness (0.58). Raykov’s reliability coefficient (Raykov, 1997) is commonly seen as more accurate than Cronbach’s alpha, and better suited for estimating the reliability of measures in Structural Equation Modeling (SEM) (Mehmetoglu and Jakobsen, 2016).

#### Insomnia

The Bergen Insomnia Scale (BIS) (Pallesen et al., 2008) contains six items that assess symptoms of insomnia based on the American Psychiatric Association’s (2000) Diagnostic and Statistical Manual of Mental Disorders-IV. The participants were asked at Wave 5 to indicate how many days a week (0–7) during the last month they have struggled with six specific symptoms of insomnia. The first three items measure sleep onset, sleep maintenance, and early morning wakening insomnia, respectively. The last three items refer to not feeling adequately rested, experiencing daytime impairment due to poor sleep and being dissatisfied with current sleep. The scale can be used either as a continuous measure, where higher scores indicate more symptoms of insomnia, or as a dichotomous measure, where the diagnostic criteria of insomnia are met with a score of >2 on one or more of the first four items (nocturnal symptoms) and a score of >2 on one or more of the last two items (daytime symptoms). In the present study, we used the continuous measure of insomnia. The scale has good convergent and discriminative validity, and satisfying psychometric properties (Pallesen et al., 2008). The Raykov’s reliability coefficient (RRC) for insomnia was high (0.80).

#### Anxiety and Depression

Anxiety and depression were assessed at Wave 5 and 6 with the Hospital Anxiety and Depression Scale (HADS) (Zigmond and Snaith, 1983). The instrument consists of 14 items, equally divided on the anxiety and depression sub-scales. The participants were instructed to indicate the way they felt during the last week prior to measurement. The responses were given on a 4-point scale which is customized to each item. The anxiety scale contains items that measures symptoms related to generalized anxiety and panic attacks (e.g., “I get sudden feelings of panic”), while the depression scale generally covers anhedonia (e.g., “I feel cheerful”). HADS is found to perform well in terms of assessing symptoms and severity of anxiety and depression in the general population, and possesses a good balance between sensitivity and specificity for the separate constructs (Bjelland et al., 2002). A validated Norwegian language version of the instrument was used (Mykletun et al., 2001). Raykov’s reliability coefficient (RRC) was high for both anxiety and depression (0.80).

### Statistical Analyses

The statistical analyses were performed in Stata, version 15. To test if insomnia functioned as a mediator between personality and future symptoms of anxiety and depression, we performed structural equation modeling (SEM) analysis with maximum likelihood including missing values estimation (MLMV). The analysis for each outcome (insomnia, anxiety, and depression) included all observed and latent variables in a single step. A correlation between the residuals of the endogenous latent variables anxiety and depression was included, making the model equivalent to multivariate regression. The *medsem* command in Stata (Mehmetoglu, 2018) estimated the indirect effects in the mediation model. This is a considerably modified and improved version of the Baron and Kenny’s (1986) approach by Iacobucci et al. (2007). We used the Monte Carlo method to test the significance of the indirect effects, as it can be considered more suitable than Sobel’s test in overcoming challenges with possible non-normality (Mehmetoglu, 2018). The *medsem* Stata package for statistical mediation offers a complete and thorough mediational model where both observed and latent variables can be included (Mehmetoglu, 2018), as was the case in the present study. An additional SEM-analysis was conducted to investigate if the findings remained significant when adjusting for anxiety and depression at Wave 5.

## Results

We performed a dropout analysis, investigating the difference between respondents and non-respondents at Wave 6. Chi square and independent *t*-tests with Bonferroni correction for family wise error rate (Dunn, 1961) tested the difference between respondents in Wave 4 and 5 on anxiety, depression, insomnia, age (measured at Wave 5), and personality (measured at Wave 4). None of the differences turned out significant.

The relationship between the variables, means and standard deviations are presented in Table 1. Anxiety and depression were strongly correlated (*r* = 0.62, *p* < 0.001), and both were moderately correlated with insomnia (*r* = 0.36, *p* < 0.001). Neuroticism had moderate to strong correlations to insomnia (*r* = 0.32, *p* < 0.001), anxiety (*r* = 0.50, *p* < 0.001), and depression (*r* = 0.41, *p* < 0.001).

**TABLE 1:** Means, standard deviations, Raykov’s reliability coefficients, and Pearson correlation coefficients for all continuous variables (*N* = 1506–1516).

	1	2	3	4	5	6	7	8
1. Neuroticism	(0.73)							
2. Extraversion	$-0.16^{*,**}$	(0.78)						
3. Conscientiousness	$-0.24^{*,**}$	$0.14^{*,**}$	(0.58)					
4. Insomnia	$0.33^{*,**}$	$-0.06^{*}$	$-0.11^{*,**}$	(0.80)				
5. Anxiety	$0.50^{*,**}$	$-0.08^{**}$	$-0.17^{*,**}$	$0.36^{*,**}$	(0.80)			
6. Depression	$0.41^{*,**}$	$-0.16^{*,**}$	$-0.17^{*,**}$	$0.36^{*,**}$	$-0.62^{*,**}$	(0.82)		
7. Age^a^	$-0.12^{*,**}$	$-0.05^{**}$	$0.07^{**}$	0.01	-0.05	$0.06^{*}$	−	
8. Gender	$0.10^{*,**}$	0.06	$0.14^{*,**}$	0.01	0.00	0.02	0.05	−
Mean	2.79	3.54	4.20	$12.92{}^{b}$	$9.16{}^{b}$	$5.20{}^{b}$	38.16	1.91
SD	0.86	0.80	0.63	8.30	6.93	5.75	8.45	0.28

*p < 0.05, ^∗∗^*p* < 0.01, ^∗∗∗^*p* < 0.001. ^a^At Wave 6. ^b^Sum score.

### Model Fit Indices

The model fit was χ^2^ = 2373.43, *p* < 0.001. The root mean squared error of approximation (RMSEA) was 0.04, 90% CI (0.03, 0.04). Values below 0.08 are considered acceptable fit, while values below 0.05 are considered good fit. The comparative fit index (CFI) and the Tucker-Lewis index (TLI) is recommended to be at or above 0.90 in order to indicate acceptable model fit (Acock, 2013). In this model, the CFI was 0.91, and the TLI was 0.90, indicating acceptable fit.

### Hypothesis Testing

**FIGURE 2:**
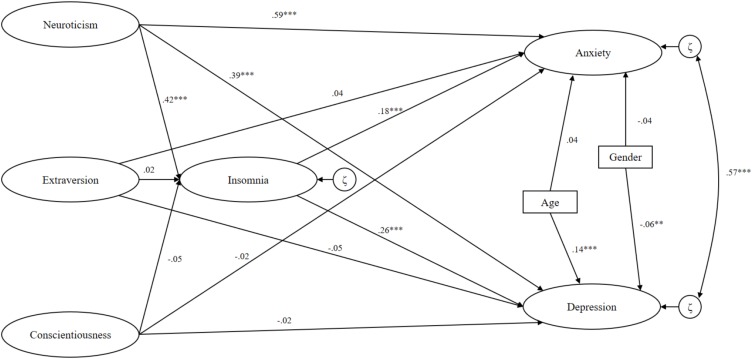
Latent paths between variables in the structural model.

The structural model showing the direct and indirect relations is presented in Figure 2, while the standardized path coefficients and associated statistics are presented in Table 2. Neuroticism predicted insomnia (β = 0.42, *p* < 0.001), anxiety (β = 0.59, *p* < 0.001), and depression (β = 0.39, *p* < 0.001). Extraversion and conscientiousness measured at Wave 4 were not significant predictors of the endogenous variables measured at Wave 6. Older individuals were more prone to depression (β = 0.14, *p* < 0.001), but not anxiety, while men were slightly more prone to anxiety compared to women (β = −0.05, *p* = 0.043). Insomnia had a significant direct relation to both anxiety (β = 0.18, *p* < 0.001) and depression (β = 0.26, *p* < 0.001). Insomnia also partially mediated a proportion the relationship between neuroticism and anxiety and depression. In addition to the direct association, neuroticism had an indirect association to anxiety (β = 0.08, *p* < 0.001) and depression (β = 0.11, *p* < 0.001) through insomnia. Thus, 11% of the relationship between neuroticism and anxiety, and 22% of the relationship between neuroticism and depression was explained by insomnia. The residuals of anxiety and depression were strongly associated with each other (β = 0.57, *p* < 0.001), meaning that the two endogenous variables shared about 30% of their variance that was not already explained by the exogenous variables.

**TABLE 2:** Standardized path coefficients in the structural model showing direct and indirect relationships between personality, insomnia, anxiety, and depression (*N* = 2962).

		Direct relations			Indirect relations through insomnia			
Outcome	Predictor	β	*SE*	95% CI	β	*SE*	95% CI	RIT
Insomnia	Neuroticism	0.42***	0.03	(0.36, 0.48)				
	Extraversion	0.02	0.03	(−0.03, 0.08)				
	Conscientiousness	-0.05	0.04	(−0.12, 0.02)				
Anxiety	Neuroticism	0.59***	0.03	(0.53, 0.66)	0.08***	0.01	(0.05, 0.10)	0.11
	Extraversion	0.04	0.03	(−0.01, 0.09)	0.01	0.01	(−0.01, 0.02)	0.10
	Conscientiousness	-0.02	0.03	(−0.09, 0.05)	-0.01	0.01	(−0.02, 0.00)	0.30
	Insomnia	0.18***	0.03	(0.12, 0.24)				
	Age	0.04	0.02	(−0.01, 0.08)				
	Gender^a^	-0.04	0.02	(−0.08, 0.01)				
Depression	Neuroticism	0.39***	0.04	(0.32, 0.46)	0.11***	0.02	(0.08, 0.14)	0.22
	Extraversion	-0.05	0.03	(−0.10, 0.01)	0.01	0.01	(−0.01, 0.02)	0.15
	Conscientiousness	-0.02	0.04	(−0.09, 0.05)	-0.01	0.01	(−0.03, 0.01)	0.39
	Insomnia	0.26***	0.03	(0.20, 0.31)				
	Age	0.14***	0.02	(0.10, 0.18)				
	Gender^a^	-0.06**	0.02	(−0.11, −0.02)				

The covariance of the error terms of anxiety and depression was 0.57. RIT, ratio of indirect to total effect, CI, confidence interval. ^a^Male = 1, female = 2. *p < 0.05, ^∗∗^*p* < 0.01, ^∗∗∗^*p* < 0.001.

When adjusting for earlier symptoms of anxiety and depression at Wave 5 (anxiety and depression were not assessed at Wave 4 in the current study), several changes in the structural model were observed. The direct relation between neuroticism and anxiety was reduced (β = 0.26, *p* = 0.001), but still significant. The direct relation between insomnia and anxiety (β = 0.01), and the indirect relation between neuroticism and anxiety through insomnia (β = 0.01) was no longer significant. The direct relation between neuroticism and depression (β = 0.15, *p* = 0.004), between insomnia and depression (β = 0.06, *p* = 0.046) and the indirect relation between neuroticism and depression through insomnia (β = 0.03, *p* = 0.048) was considerably weakened, but still significant. In all, 18% of the relationships between neuroticism and depression was mediated by insomnia in the adjusted model. The fit statistics of this model indicated poorer fit than the unadjusted model, RMSEA = 0.04, 90% CI (0.04, 0.04), CFI = 0.84, TLI = 0.83, χ^2^ = 6608.65, *p* < 0.001. The stability coefficient (r) of HADS A (anxiety) and HADS D (depression) between Wave 5 and Wave 6 were 0.71 and 0.64, respectively (*p* < 0.001).

## Discussion

Results from the present study showed that neuroticism and insomnia had significant direct and indirect relations to future levels of anxiety and depression. Extraversion and conscientiousness had no significant association to future symptoms of insomnia, anxiety or depression. In addition, neuroticism was the only personality trait that could predict future symptoms of insomnia. Insomnia was a significant predictor of both anxiety and depression, but the association was considerably weaker compared to the relationship between neuroticism and symptoms of anxiety and depression. This finding indicates that neuroticism is a stronger and more robust predictor of mental illness than insomnia. Insomnia was a significant mediator between both neuroticism and anxiety, and neuroticism and depression, meaning that neuroticism had an indirect relation to symptoms of anxiety and depression through insomnia. When adjusting for previous symptoms of anxiety and depression, the effect of insomnia as a mediator only remained significant in the relationship between neuroticism and depression. Even though insomnia could explain 18% of the relation, the size of the mediating effect was small. In addition, the effect size of the direct relation between neuroticism and depression was also reduced compared to the un-adjusted model.

### Direct Associations Between Personality, Insomnia, and Anxiety and Depression

Neuroticism predicted future symptoms of insomnia, and the first hypothesis was therefore partly supported. This finding supports the notion that neuroticism is a stable predictor for insomnia, in line with previous research (Duggan et al., 2014; Harvey et al., 2014; Hintsanen et al., 2014). In turn, insomnia predicted future symptoms of both anxiety and depression. This finding is also supported by relevant previous studies (Neckelmann et al., 2007; Baglioni et al., 2011). Consistent with earlier research (Bienvenu and Stein, 2003; Bienvenu et al., 2004; Klein et al., 2011) the results further showed that neuroticism was a significant predictor of future symptoms of anxiety and depression. No significant direct associations were found between extraversion and conscientiousness and anxiety and depression. The second hypothesis was therefore only partly supported. In sum, neuroticism was the only personality trait that predicted future symptoms of insomnia, anxiety and depression. This finding is consistent with previous studies where neuroticism is one of the most stable predisposing personality factor of negative sleep and mental health outcomes (Bienvenu et al., 2004; van de Laar et al., 2010; Duggan et al., 2014; Watson and Naragon-Gainey, 2014). Neuroticism may be at the core of internalizing psychopathology, e.g., anxiety and depression, and a necessary factor in structural theories regarding these disorders (Griffith et al., 2010).

### Insomnia as a Mediator Between Personality and Symptoms of Anxiety and Depression

Insomnia mediated parts of the positive relationship between neuroticism, anxiety and depression, and accounted for 11% of the relationship between neuroticism and anxiety, and 22% of the relation to depression. No significant indirect associations were found regarding extraversion or conscientiousness. When adjusting for anxiety and depression at Wave 5, insomnia remained a partial mediator in the relation between neuroticism and depression only. However, this result was marginally significant, and the small effect size demonstrates that the indirect association explain substantially less of the relation than the direct association between neuroticism and depression. Hence, the mediating effect should be interpreted with caution. Further, the direct effect insomnia had on anxiety was no longer significant when controlling for anxiety at Wave 5, which is in contrast to previous research where insomnia seem to be a precursor to anxiety, but not to depression (Vedaa et al., 2016b). However, Johnson et al. (2006) found that prior insomnia was associated with the onset of depression, but not anxiety disorders. These findings underline the current inconclusiveness when it comes to the relation between insomnia and anxiety, and both direct and indirect associations between the two concepts are in need of further investigation. The relationship between insomnia and depression was stronger than the relationship between insomnia and anxiety in the present study, and the findings suggest that insomnia is more important in the development of depression than anxiety. However, anxiety had a stronger relationship to neuroticism than depression, and symptom level of anxiety may hence represent a more dispositional tendency, consistent with other large scale studies using HADS over a 5 to 8-year time-span (Langvik and Hjemdal, 2015). In the present study, the time-span was 1 year, and the high stability of the symptom-level of HADS is a plausible explanation for the lack of influence of insomnia on the relation between neuroticism and anxiety. More research on insomnia as a mediator in the relationship between neuroticism and anxiety is necessary before any conclusions can be made. Hypothesis three, stating that insomnia will mediate the relation between personality traits and symptoms of anxiety and depression, was partly supported, although this was only true for symptoms of depression. This addresses the importance of separating the two outcomes, despite high comorbidity and the possible shared etiology in terms of neuroticism.

The findings in the present study can explain parts of the pathway between neuroticism and symptoms of anxiety and depression. In addition to the increased vulnerability for affective disorders caused by neuroticism, the trait can also contribute to depression and anxiety through its impact on and relation to insomnia (Gurtman et al., 2014). Even though the specific role of personality in the etiology of insomnia is not clear (van de Laar et al., 2010), the negative characteristics of neuroticism can affect several aspects of sleep through negative emotionality and heightened general vulnerability and arousal. This is indicated in our results, where neuroticism at Wave 4 was positively related to insomnia 1 year later at Wave 5. This is also supported by previous research showing that a low score on neuroticism at baseline was related to better sleep quality over time (Stephan et al., 2018). Patients with insomnia have a tendency to be overly concerned and to impose strain on themselves (Vincent and Walker, 2000; de Azevedo et al., 2009), which are characteristics related to neuroticism (Dunkley et al., 2012). In turn, this can affect sleep onset, sleep duration, awakenings and sleep quality of individuals high in neuroticism, and through this contribute to insomnia and mental illness.

Furthermore, sleep deprivation may cause elevated negative affect as a response to mild stressors (Minkel et al., 2012). Individuals suffering from insomnia may experience sleep deprivation as this disorder is characterized by difficulty initiating or maintaining sleep and waking up too early (American Psychiatric Association, 2013). Lack of sleep can make the individual more vulnerable to experience symptoms of anxiety and depression. Sleep is considered as an imperative for health (Luyster et al., 2012), and sleep deprivation can have deleterious effects on mental health in particular (Roberts et al., 2009). The finding highlights that the direct association between neuroticism and affective disorders might have been somewhat overestimated in previous literature, meaning that possible mediators (e.g., insomnia) can have been overlooked. Even so, the mediating effect size of insomnia in the adjusted model were close to zero, demonstrating that insomnia only may function as a weak partial mediator. Further, the direct relation between neuroticism and anxiety and depression remained substantial after the indirect effect had been accounted for.

### Strengths and Limitations

A limitation of the present study was the use of the MINI-IPIP to measure personality traits of the Five-factor model (FFM). In large-scale surveys, short inventories are preferred, although few items often come with the cost of reduced internal consistency (Soto and John, 2017), as observed in regards to conscientiousness in the present study. Despite its limitations, MINI-IPIP has proven to be a useful and effective tool to measure personality according to the FFM (Donnellan et al., 2006). The study followed a longitudinal design in a large homogenous sample of nurses. However, as personality traits only were measured one time, the study can be characterized as only partly longitudinal which limits the use of longitudinal structural equation modeling. Still the predictive value of the findings is highly relevant for employees in the nursing profession. The response rate at Wave 1 was quite low (38%), and only nurses participating in the first wave were invited to the following data collections. The population consisted of nurses only, and further, a selection bias was probably present. This reduces the possibility of generalization to other occupational groups. However, previous studies also point out that a response rate of around 35–40% is common and acceptable in organizational studies (Baruch and Holtom, 2008). In addition, the response rates in the follow-up waves included in the study were high, between 69 and 76%. The sample consisted of 91% females, and exclusively nurses, so generalization to more male dominated occupations may be difficult. Still, the female preponderance of the present study represents the gender distribution of nurses well, and the results can likely be generalized to other nursing and similar female-dominated populations.

### Implications and Suggestions to Further Research

Our findings highlight the importance of an integrated approach to research on the topic, and the need for taking bidirectional and circular relations into account. The extensive research on nurses, especially related to sleep, health, and shift work (Geiger-Brown and Lipscomb, 2010; Adams et al., 2018) reflects the challenges this profession face on a daily basis. A work life consisting of daily stressors in terms of emotional and physical demands, lack of sleep, time pressure, and a heavy work load have potentially deleterious effects on the mental health of a significant number of nurses (Mark and Smith, 2012). The present study demonstrated that some individuals, due to their levels of certain personality traits, e.g., neuroticism, may be particularly vulnerable to these negative work factors. This knowledge can be applied in both prevention and clinical treatment of sleep- and affective disorders, and thereby contribute to more personalized measures and treatments. As the majority of the participants in the present study were females, there is a need to investigate the relations and hypotheses in other occupations. This includes more gender equal or male dominated professions to strengthen the generalizability of the findings. As highlighted by Klein et al. (2011), there is a profound need for the development of explanatory models to strengthen the understanding of how personality and psychopathology are connected. To take the field of research one step further, more attention should be paid to the mediating variables that can explain how and why certain personality traits and mental illness are related.

## Conclusion

We found a direct relationship between neuroticism and insomnia, anxiety and depression in line with previous studies. In addition, the present study demonstrated an indirect relationship between neuroticism and anxiety and depression through the association between neuroticism and insomnia. However, when adjusting for previous symptoms of anxiety and depression, insomnia still partly mediated the relationship between neuroticism and future symptoms of depression, but not anxiety. No significant relationships were found between conscientiousness, extraversion and symptoms of anxiety and depression. In sum, the results indicate that among the Five Factor Model personality traits, only neuroticism seemed to be a predictor for anxiety and depression in the present study. However, as the sample mainly consisted of female nurses, generalization to other and more male dominated professions is challenging. In conclusion, insomnia may act as a partial mediator between neuroticism and anxiety and depression, but the direct relation between neuroticism and anxiety and depression is considerably stronger than the indirect association through insomnia.

## Ethics Statement

The project was approved by the Regional Committee for Medical Research Ethics (REK) of Western Norway (REK-West, No. 088.08).

## Author Contributions

IS-L and TS conceived the present idea. TS was in charge of the overall execution and planning of the present study, contributed to all parts of the article and wrote the first draft of the manuscript, edited all drafts, and developed the introduction, hypotheses, methods, and discussion together with IS-L and EL. HK performed the statistical analyses and wrote the first draft of the results. EL and IS-L contributed to all parts of the study, the introduction and discussion in particular, and edited all drafts. SP, BB, SW, and BM designed the SUSSH-study, collected the data, and edited the drafts of the manuscript. All authors contributed to the final work and revisions on the manuscript.

## Conflict of Interest Statement

The authors declare that the research was conducted in the absence of any commercial or financial relationships that could be construed as a potential conflict of interest.
